# Effect of Vitamin D Receptor Activators on Glomerular Filtration Rate: A Meta-Analysis and Systematic Review

**DOI:** 10.1371/journal.pone.0147347

**Published:** 2016-01-26

**Authors:** Qian Zhang, Ming Li, Tiansong Zhang, Jing Chen

**Affiliations:** 1 Division of Nephrology, Huashan Hospital and Huashan Hospital North, Shanghai Medical College, Fudan University, Shanghai, China; 2 Department of Respiratory Medicine, Shanghai Tenth People's Hospital Affiliated to Tongji University, Shanghai, China; 3 Department of TCM, Jing’an District centre hospital of Shanghai, Shanghai, China; 4 Division of Nephrology, Huashan Hospital, Shanghai Medical College, Fudan University, Shanghai, China; Ichan School of Medicine at Mount Sinai, UNITED STATES

## Abstract

**Background:**

Vitamin D receptor activators (VDRAs) can protect against mineral bone disease, but they are reported to elevate serum creatinine (SCr) and may also reduce glomerular filtration rate (GFR).

**Methods:**

We conducted a systematic review and meta-analysis of randomized clinical trials (RCTs) to evaluate the effect of VDRAs on kidney function and adverse events. MEDLINE, EMBASE, the Cochrane Controlled Trials Register were searched for RCTs that evaluate vitamin D receptor activators (alfacalcidol, calcitriol, doxercalciferol, falecalcitriol, maxacalcitol and paricalcitol) up to March 2015.

**Results:**

We included 31 studies, all of which were performed between 1976 and 2015, which enrolled 2621 patients. Patients receiving VDRAs had lower eGFR (weighted mean difference WMD -1.29 mL/min /1.73 m^2^, 95% CI -2.42 to -0.17) and elevated serum creatinine (WMD 7.03 μmol/L, 95% CI 0.61 to 13.46) in sensitivity analysis excluding studies with dropout rate more than 30%. Subgroup analysis of the 5 studies that not use SCr-based measures did not indicated lower GFR in the VDRAs group(WMD -0.97 mL/min/1.73 m2, 95% CI -4.85 to 2.92). Compared with control groups, there was no difference in all-cause mortality (relative risk RR 1.41, 95% CI 0.58 to 3.80), cardiovascular disease (RR 0.84, 95% CI 0.42 to 1.71), and severe adverse events (RR 1.15, 95% CI 0.75 to 1.77) for the VDRAs groups. Episodes of hypercalcemia (RR 3.29, 95% CI 2.02 to 5.38) were more common in the VDRAs group than in the control group.

**Conclusions:**

Administration of VDRAs increased serum creatinine levels. Subgroup analysis of studies that did not use SCr-based measures did not indicate a lower GFR in the VDRA group. Future studies with non-SCr-based measures are needed to assess whether the mild elevations of serum creatinine are of clinical significance.

## Introduction

Vitamin D is synthesized in the skin or ingested in the diet. It is subsequently converted to the active metabolite 1,25(OH)2 vitamin D [1]. The consequences of vitamin D deficiency are secondary hyperparathyroidism and bone loss, leading to osteoporosis and fractures, mineralization defects, causing falls and fractures [2]. Therefore, vitamin D receptor activators (VDRA), such as calcitriol, paricalcitol, or doxercalciferol, have been developed to treat osteoporosis, chronic kidney disease-mineral and bone disorder (CKD-MBD), and can also reduce podocyte injury, modulate immune responses, and improve insulin sensitivity [3–6].

The Vitamin D Receptor Activator for Albuminuria Lowering (VITAL) Study demonstrated that addition of paricalcitol to an inhibitor of the rennin-angiotensin-aldosterone system (RAAS) safely lowered residual albuminuria in patients with diabetic nephropathy [7]. However, patients given high-dose paricalcitol (2 μg daily) experienced significant declines in estimated glomerular filtration rate (eGFR). Although the eGFR values of these patients returned toward baseline after drug withdrawal, this raises a concern that VDRAs may lead to nephrotoxicity in CKD patients.

In 1978, Christiansen et al. reported that deterioration of renal function limited the use of calcitriol in non-dialysis patients with chronic renal failure [8]. More recently, Agarwal et al. indicated that short-term paricalcitol increased the level of serum creatinine (SCr), but it did not influence eGFR [9]. The Paricalcitol Capsule Benefits in Renal Failure–Induced Cardiac Morbidity (PRIMO) trial measured the effects of paricalcitol on left ventricular mass in patients with eGFRs of 15 to 60 mL/min/1.73 m^2^ (calculated by creatinine-based equations). This study also reported a small but significant reduction of eGFR in the paricalcitol group [10].

Concerns about the possible acceleration of kidney function decline have long limited the use of VDRAs. Previous meta-analysis and systematic reviews confirmed that active vitamin D analogs suppress parathyroid hormone (PTH) and reduce proteinuria in CKD patients without increasing the risk of adverse events [11,12]. However, these studies did not include non-CKD patients or evaluate the changes in GFR and adverse events as primary endpoints. The effects of VDRAs on kidney function remain uncertain. Thus, we performed a systematic review and meta-analysis from randomized clinical trials (RCTs) that investigated the effect of VDRAs on GFR and other hard endpoints in both CKD and non-CKD patients. The aim of the study is to find out whether VDRAs reduce eGFR, increase SCr or have adverse reactions, and to comprehensive understand the role of VDRAs in patients.

## Methods

### Data sources and searches

We performed a systematic review of the available literature in accordance with the PRISMA guidelines [13]. This entailed searches of MEDLINE, EMBASE, and the Cochrance Controlled Trials Register up to March 2015 for relevant keywords, including all spellings of vitamin D receptor activators (alfacalcidol, calcitriol, doxercalciferol, falecalcitriol, maxacalcitol and paricalcitol), and serum creatinine (SCr) or cystatin C or creatinine clearance (CCr) or glomerular filtration rate (GFR) or estimated glomerular filtration rate (eGFR). We excluded studies in which patients were given native vitamin D (ergocalciferol or cholecalciferol). When an abstract did not contain such data, but the presence of such data was expected in the full-text paper, the full-text paper was screened as well. We also searched for these terms in the abstracts of conference proceedings of the American Society of Nephrology and the European Renal Association-European Dialysis and Transplant Association. The references of all included trials and review articles were screened for additional studies. If necessary, the authors of the clinical trials were asked to provide additional data.

### Study selection

Study reports were included if they: *(i)* were RCTs; *(ii)* enrolled adult subjects (CKD, osteoprosis, patients undergoing organ transplantation or any other reason receiving VDRA treatment) who received a VDRA or control treatment (placebo or no treatment); *(iii)* provided data on SCr, cystatin C, CCr, GFR, or eGFR; and *(iv)* were clinical trials regardless of publication status (published, conference proceedings, or unpublished), trial year, and language of publication. Two individuals (Q.Z. and M.L.) independently inspected each reference and applied the inclusion criteria. If data on the same patient population were in more than one study, the most recent study was included. For possibly relevant articles or in cases of disagreement, each author inspected the full article independently. The primary outcome was kidney function (eGFR and SCr) and the secondary outcomes were complications (death, cardiovascular disease [CVD], end stage renal disease [ESRD], adverse events, severe adverse events, and hypercalcemia). However, there was no registration number for this systematic review.

### Data extraction and risk of bias

We developed a standard data form to record the following for each study: all authors, publication date, type of study, sample size, number of patients (in total and by treatment assignment), number of patients excluded, number of patients observed, number of patients lost to follow-up, population characteristics (age, sex, and menopausal status), stage of CKD, presence of diabetes, use of angiotensin converting enzyme inhibitor/angiotensin receptor blocker (ACEI/ARB), and laboratory results at randomisation. For each RCT, we also recorded the independent randomisation centre, type of blinding, random allocation, adequate concealment of allocation, intention to treat, withdrawal or dropout rate, and trial intervention. Two individuals (Q.Z. and M.L.) independently extracted data from all primary studies that fulfilled the inclusion criteria. Disagreements were resolved by consensus.

The same reviewers independently assessed the risk of bias in the included studies without blinding to authorship or journal name, to assess the risk of bias in sequence generation, allocation concealment, blinding, attrition, selection, and other areas. Studies were rated as having a high risk for bias when at least one of these was rated as “high risk”.

### Data synthesis and analysis

For continuous variables, we pooled data by calculation of weighted mean differences (WMDs) of the groups so that more weight was given to superior studies. Means and SDs for changes from baseline in each group were obtained for all continuous variables. When these were not available, they were calculated from data provided by the investigators, from figures, or by recalculation from other effect estimates and dispersion measures [14]. We also computed correlation coefficients from one study [15], and calculated standard deviations for changes from baseline using methods described in *Cochrane Handbook for Systematic Reviews of Interventions (ver*. *5*.*1*.*0)* [14]. Dichotomous data were compared using relative risk (RR) and risk difference (RD) and 95% confidence intervals (CIs) were calculated for each estimate and presented in forest plots.

We combined our studies using the DerSimonian and Laird random effects model, because this method partially accounts for variability within and between studies [16]. We calculated the *I*^*2*^ statistic to assess heterogeneity among studies, and classified values less than 50% as minimal, 50–75% as moderate, and >75% as substantial [14,17].

To assess clinical heterogeneity based on characteristics of study population and interventions, we performed subgroup analyses of: *(i)* patients given different VDRAs; *(ii)* patients with different baseline eGFRs (<60 mL/min/1.73 m^2^ *vs*. ≥60 mL/min/1.73 m^2^); We performed a sensitivity analysis on kidney function outcomes by excluding studies with a high risk of bias for one or more key domains using the Cochrane Collaboration tool for assessment of the risk of bias [18]. Further analyses were performed by excluding studies that had a dropout rate more than 30%. Meta-regression was undertaken to examine the effect of gender and hypercalcemia rate on the associations between VDRAs therapy and eGFR changes.

The potential presence of publication bias was examined by inspection of funnel plots and by the Egger linear regression test [19]. Stata (ver. 11.0) software that incorporated the updated metan meta-analysis package was used for all statistical analyses [20]. All statistical tests were two sided and a *p*-value less than 0.05 was considered significant.

## Results

### Study selection

**Fig 1:**
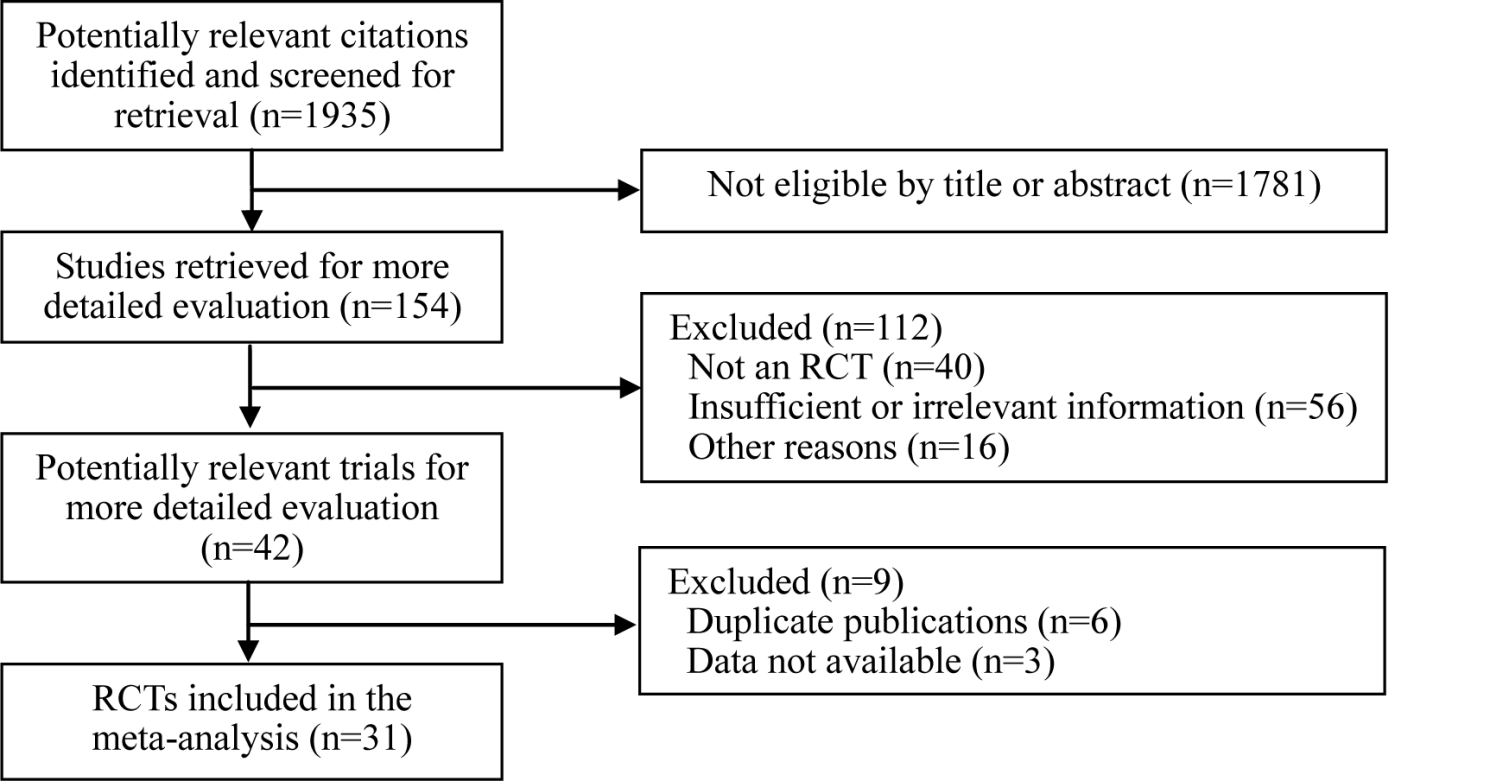
Procedure used for the trial selection. RCT = Randomized controlled trial.

We performed a systematic review of the available literature in accordance with the PRISMA guidelines (see S1 Table). Fig 1 shows the procedure used for selection of clinical studies that examined the effect of VDRAs on GFR. We identified 1935 articles in the initial search, and excluded 1781 of these by screening the titles and abstracts. Among the remaining 154 articles, 123 were excluded for reasons indicated in Fig 1. The 31 included studies were performed between 1976 and 2014[7,10,15,21–48], and enrolled a total of 2621 patients. None of the reviewed conference abstracts met the inclusion criteria, so these were excluded from analysis. Multiple publications were excluded from the count of included studies because these were secondary publications of previous reports; however, any relevant and unique results from these secondary publications were extracted and included.

### Study characteristics

We included studies which enrolled patients with CKD, transplant recipients, postmenopausal osteoporosis patients and elderly women. Table 1 summarizes the characteristics of the included studies and participants. These studies compared patients treated with a VDRA (alfacalcidol, calcitriol, doxercalciferol, or paricalcitol) with patients given a placebo or no treatment. None of RCTs of maxacalcitol or falecalcitriol met the inclusion criteria. Seventeen studies enrolled patients with CKD [7,10,15,21–22,24,26,28–29,33,37–40,45,47–48]; eight studies enrolled[23,25,30–32,34–35,44] transplant recipients, seven[23,25,30–32,35,44] of which were renal transplant recipients; five studies[27,36,41–42,46] enrolled postmenopausal osteoporosis patients; and one study[43] enrolled elderly women. Twenty-three [7,10,15,21–22,24,27–29,31,33–36,38,40–48]of the 31 included studies compared VDRAs with placebo, and eight studies [23,25–26,30,32,37,39,44]compared calcitriol with no treatment. The studies varied in sample size (13 to 415 patients), mean patient age (31.5 to 70.7 years), and treatment duration (1 month to 3 years).

**Table 1:** Characteristics of studies included in the meta-analysis.

Study	GFR estimation method	Baseline Disease	eGFR VDRAs group (ml/min/1.73 m^2^)		eGFR Control group(ml/min/1.73 m^2^)		SCr VDRAs (umol/L)		SCr Control (umol/L)		Mean Age (years)	Sex (Male %)	Diabetes Mellitus (%)	ACEI/ARB Use (%)	VDRAs group (n)	Control group (n)	Follow-up	Risk of bias score	Jadad score ^e^
			Baseline	After treatment	Baseline	After treatment	Baseline	After treatment	Baseline	After treatment									
Alborzi 2008[24]	iothalamate	CKD	1μg/d 47.5±9.42μg /d 47.4±12.7	1μg/d -3.2(-3.1 to-2.9)^c^ 2μg /d 6.9(-1.4 to 15.2)^c^	44.0±12.0	5.3(-3.1 to 13.7)^c^	NA	NA	NA	NA	69.5±10.2	83	70.8	100	Paricalcitol 1μg/d (8) 2μg /d (8)	Placebo (8)	1mo	Low	5
Aloia 1988[46]	CrCl	Postmenopausal osteoporosis	69.4±6.9	101±12.0^a^	58.7±6.1	125±21.4^a^	NA	NA	NA	NA	64.9±1.7(placebo)64.1±1.5(VDRA)	0	0.0	NA	Calcitriol 0.8 ug /d (12)	Placebo (15)	24mo	High	4
Amer 2013[25]	MDRD equation, iothalamate or CrCl	Renal transplantation	45.0±15.4	51.2±15.4	45.3±10.0	52.7±14.1	NA	NA	NA	NA	48.1±10.1	66	18.0	NA	Paricalcitol 2μg /d (51)	No treatment (49)	1yr	High	3
Baker 1989[47]	CrCl	CKD	34.7±14	31.4±16.3	44.7±13.1	40.2±14.3	0.240±0.071(mmol/L)	0.286±0.108 (mmol/L)	0.220±0.103 (mmol/L)	0.242±0.166(mmol/L)	52.5(31–64)	54	0.0	NA	Calcitriol 0.25–0.5 ug /d (8)	Placebo (8)	52wk	Unclear	4
Coburn 2004[48]	CrCl	CKD3-4	34.2±2.7	30.0±2.9	36.4±3.2	33.9±3.3	3.02±0.97(md/dl)	NA	3.06±0.83 (md/dl)	NA	65.0±12.1(placebo) 64.1±12.6(VDRA)	82	NA	NA	Doxercalciferol 1.0 ug/d (27)	Placebo (28)	24wk	High	5
Coyne 2006[15]	MDRD equation	CKD3-4	23.9±0.90	21.4±0.99	23.4±0.85	21.9±0.93	2.92±0.092(mg/dl)	3.33±0.138(mg/dl)	2.94±0.086 (mg/dl)	3.30±0.129(mg/dl)	61.8±12.4(placebo) 63.6±13.2(VDRA)	68	58.5	69.0	Paricalcitol 1.3 to 1.4μg /d (107)	Placebo (113)	24wk	Unclear	5
Cueto-Manzano 2000[44]	NA	Renal transplantation	63.9(45–113)^b^	32.4(27–51)^b^	63(48–90) ^b^	27.8(23–25) ^b^	1.5±0.3(mg/dl)	1.7±0.3(mg/dl)	1.4±0.2(mg/dl)	1.5±0.2(mg/dl)	44.3±9.4(placebo) 51.7±11.9(VDRA)	53	NA	NA	Calcitriol 0.25 ug/d (16)	No treatment (14)	1yr	High	2
De Boer 2013[21]	CrCl	CKD3-4	38.5±11.6	-6.3(-13.5 to 0.9^c^	40.4±12.3	-0.2(-6.2 to 5.9) ^c^	NA	NA	NA	NA	65.8±11.6	91	0	82.0	Paricalcitol 2μg /d 11)	Placebo (11)	24wk	Unclear	5
De Sevaux 2002[32]	CrCl, CG equation	Renal transplantation	NA	65 ± 18	NA	64 ± 19	841 ± 289(μmol /L)	133 ± 39(μmol /L)	820 ± 209(μmol /L)	126 ± 35(μmol /L)	49±14(placebo) 46±12(VDRA)	59	6.3	NA	Calcitriol 0.5ug/48h (65)	No treatment (46)	6mo	High	3
De Zeeuw 2010[7]	MDRD equation	Type 2 diabetes and albuminuria	40±15(1μg /d) 42±18(2μg /d)	-1.2(-3.8 to 1.4) ^c^ -7.6(-10.1 to -5.1) ^c^	39±17	-0.1(-2.6 to 2.4) ^c^	172±56(1μg /d) 170±63(2μg /d) (μmol /L)	NA	180±79(μmol /L)	NA	64.9±10.4	69	100	100	Paricalcitol 1μg /d (93) 2μg /d (95)	Placebo (93)	24wk	Low	5
El-Agroudy 2003[31]	NA	Renal transplantation	NA	NA	NA	NA	1.3±0.5(mg/dl)	1.4±0.4	1.3±0.3	1.5±0.4	31.6±10.7(placebo) 31.4±10.1(VDRA)	100	0	NA	Alfacalcidol 0.5ug/d (20)	Placebo (20)	1yr	Unclear	4
El-Agroudy 2005[30]	NA	Renal transplantation	NA	NA	NA	NA	1.2±0.3 (mg/dl)	1.4±0.3	1.3±0.3	1.3±0.4	31.67±10.1(placebo) 31.4±10.1(VDRA)	100	0	NA	Alfacalcidol 0.5ug/d (15)	No treatment (15)	1yr	High	4
Gallagher 1990[42]	CrCl	Postmenopausal osteoporosis	1.08±0.23(mL/s)	1.06±0.59(mL/s)	1.08±0.33(mL/s)	0.92±0.27(mL/s)	71±10(μmol /L)	74±9(μmol /L)	73±14(μmol /L)	78±21(μmol /L)	70.5±7.5(placebo) 69.1±5.9(VDRA)	0	0	NA	Calcitriol 0.62 ug/d (25)	Placebo (25)	2yr	Unclear	5
Gallagher 2007[43]	CrCl	Elderly women	50.9±0.79(CrCl<60) 80.5±0.88(CrCl>60)	50.0±1.68(CrCl<60) 78.8±1.53(CrCl>60)	50.9±0.79(CrCl<60) 80.5±0.88(CrCl>60)	49.95±2.01(CrCl<60) 83.8±1.81(CrCl>60)	NA	NA	NA	NA	72.0±0.34(CrCl<60) 71.1±0.20(CrCl>60)	0	NA	NA	Calcitriol 0.25 ug twice daily (203)	Placebo (212)	3yr	High	3
Hamdy 1995[28]	CrCl	CKD	NA	-5.7±1.0^c^	NA	-4.0±2.0^c^	263±119(μmol /L)	78.8±15.6^c^	263±127(μmol /L)	74.1±18.7^c^	51±16 (placebo) 53±15(VDRA)	61	NA	NA	Alfacalcidol 0.25ug/d initially (89)	Placebo (87)	2yr	Unclear	4
Ivarsen 2012[26]	CrCl	CKD 4	23.30±3.0	19.2±2.7	22.4±1.7	21.0±1.6	318±37(μmol /L)	436±98(μmol /L)	330±16(μmol /L)	433±98(μmol /L)	52.0 (40–66)^b^	77	0	71.0	Alfacalcidol 0.50ug/d initially (6)	No treatment (7)	6mo	High	3
Krairittichai 2012[39]	MDRD equation	Diabetic kidney disease	37.93±18.30	36.9±19.8	36.51±16.50	35.5±17.6	2.13±0.80(mg/dl)	NA	1.99±0.70(mg/dl)	NA	61.8±11.90(placebo) 59.70±8.50(VDRA)	47	100.0	57.1	Calcitriol 0.5 ug twice weekly (46)	No treatment (45)	16wk	High	2
Liu 2012[37]	MDRD equation	IgA Nephropathy	83.1±35.8	3.2(-8.1 to 1.7) ^c^	78±28.2	0.0(-4.9 to 4.9) ^c^	104.8±42.7(μmol /L)	NA	103.3±34.5(μmol /L)	NA	36.3±10.2(placebo) 35.6±10.8(VDRA)	58	0	100	Calcitriol 0.5 ug/wk (26)	No treatment (24)	48wk	High	3
Menczel 1994[27]	CrCl	Postmenopausal osteoporosis	82±27	76±17	88±28	73±17	0.9±0.2(mg/dl)	0.9±0.1(mg/dl)	0.8±0.2(mg/dl)	0.9±0.2(mg/dl)	65.6±8.0(placebo) 68.6±6.9(VDRA)	0	NA	NA	Alfacalcidol 0.25ug twice daily (24)	Placebo (42)	3yr	Unclear	2
Nordal 1988[40]	CrCl	CKD	23.5±10.1	29±11	18.3±11.2	23.4±11	398±142	403±165	469.7±146	495.5±189	47(23–71) ^b^	67	6.7	NA	Calcitriol≤0.50 ug/d (15)	Placebo (15)	8mo	Unclear	4
Ott 1989[41]	CrCl	Postmenopausal osteoporosis	1.00±0.05(mL/s)	-3.0±4^c^	1.08±0.05(mL/s)	-6.5±4 ^c^	79±2(μmol /L)	NA	76±3(μmol /L)	NA	67.1±1.2(placebo) 67.9±1.0(VDRA)	0	NA	NA	Calcitriol 0.43 ug/d (43)	Placebo (43)	2yr	Unclear	4
Pérez 2010[23]	MDRD equation	Renal transplantation	45.04±12.79	43.92±13.32	49.06±10.86	50.41±17.10	144.96±46.60(μmol /L)	146.64±59.34(μmol /L)	128.59±34.13(μmol /L)	132.18±38.06(μmol /L)	53±9 (placebo) 57±10(VDRA)	86	NA	NA	Paricalcitol 1μg /d (25)	No treatment (17)	3mo	High	2
Przedlacki 1995[33]	^99m^Tc DTPA	CKD	21.5±3.2	18.7±5.2	31.3±4.0	26.3±3.7	340.6±35.5(μmol /L)	448.5±56.4(μmol /L)	272.6±32.8(μmol /L)	401.8±103.1(μmol /L)	50.3±2.9(placebo) 49.3±3.0(VDRA)	50	0.0	NA	Calcitriol 0.25 ug/d (13)	Placebo (13)	1yr	Unclear	3
Riggs 1985[36]	CrCl	Postmenopausal osteoporosis	79±2	75±2	82±3	84±3	NA	NA	NA	NA	64.0	0	NA	NA	Calcitriol 0.50–0.75 ug/d (30)	Placebo (26)	2yr	Unclear	4
Ritz 1995[38]	NA	CKD	NA	NA	NA	NA	9.1(8.3–19.6)	7.3(4.5–26.1)	10.6(6.6–34.2)	12.8(4.2–57.9)	52(26–28) (placebo) 54(27–70) (VDRA)	48	0.0	NA	Calcitriol 0.125 ug/d (33)	Placebo (33)	1yr	Unclear	3
Rix 2004[29]	CrCl	CKD	49±20	28±4	36±13	26±5	NA	NA	NA	NA	52.5	69	NA	NA	Alfacalcidol 0.25–0.75ug/d (18)	Placebo (18)	18mo	Unclear	5
Sambrook 2000[34]	NA	Cardiac or lung transplantation	NA	NA	NA	NA	0.10±0.02	0.14±0.04	0.11±0.02 mmol/L	0.14±0.06	45.35(27–56)(placebo) 45.8(22–65) (VDRA)	72	NA	NA	Calcitriol 0.5–0.75 ug /d (44)	Placebo (21)	2y	Unclear	4
Thadhani 2012[10]	SCr-based and cystatin C-based equation	CKD	31(24–43)	-4.1±0.9 ^c^	36(26–42)	-0.1±0.7 ^c^	2.1(1.6–2.7)	NA	1.9(1.6–2.4)	NA	66±12(placebo) 64±11(VDRA)	70	57.0	82.0	Paricalcitol 2 μg/d (115)	Placebo (112)	48wk	Unclear	5
Torres 2004[35]	CrCl	Renal transplantation	71.6±24.5	83.7±30	69.2±26.6	76±30	1.39±0.4	1.37±0.3	1.4±0.5	1.3±0.4	51.1±11.9 (placebo) 46.7±12.2(VDRA)	78	25.8	NA	Calcitriol 0.5 ug/48 h (45)	Placebo (41)	1yr	Unclear	4
Tougaard 1976[45]	EDTA	CKD	11.2	-2.8±2.5^c^	13.5	-1.1±3.1^c^	NA	NA	NA	NA	20–70^d^	63	NA	NA	Calcitriol 1 ug/d (12)	Placebo (12)	11wk	Unclear	3
Wang 2014[22]	MDRD equation	CKD3-5	19.7(16.0–30.6)	-4.49(-6.51 to -2.48)	23.9(20.5–31.3)	-3.03(-5.04 to -1.01)	NA	NA	NA	NA	62.2±10.7 (placebo) 60.8±10.2(VDRA)	53	34.9	81.7	Paricalcitol 1μg/d (30)	Placebo (30)	52wk	Unclear	5

NA = not available. CKD = chronic kidney disease. eGFR = estimated glomerular filtration rate. ACEI = angiotensin-converting enzyme inhibitors. ARB = angiotensin receptor blocker. VDRA = Vitamin D receptor activation. μg = microgram. EDTA = ethylenediaminetetraacetic acid CrCl = 24hour urine creatinine clearance CG equation = Cockcroft Gault equation.

^a^Data expressed as percent change.

^b^Data expressed as median(25% to 75%)

^c^Data expressed as change from baseline.

^d^Data expressed as age range.

^e^The Jadad score is a statistical point system based on 5 components to evaluate the quality of studies: randomization, method of randomization being appropriate and described, double-blinding, double-blinding being appropriate and described, and description of withdrawal and dropouts.

### Risk of bias

Nine studies [7,15,22,24–25,30,37,42,48] described the methods used for random sequence generation and eight studies [7,15,21,24,30–31,37,42] described the methods used for allocation concealment. Fourteen studies [7,10,15,21–22,24–26,28,35,37–39,43] described all expected outcomes, but eighteen studies [15,23,26,28–29,31,33,35–37,39–42,44,46–48] did not describe whether the analyses were by intention-to-treat. Overall, the risk of bias was high for 11 studies [23,25–26,30,32,37,39,43–44,46,48]. Eight of these studies [23,25–26,30,32,37,39,44] did not blind the participants or study personnel and four studies [32,43,46,48] described incomplete outcome data (see S2 and S3 Tables).

### eGFR outcome

**Fig 2:**
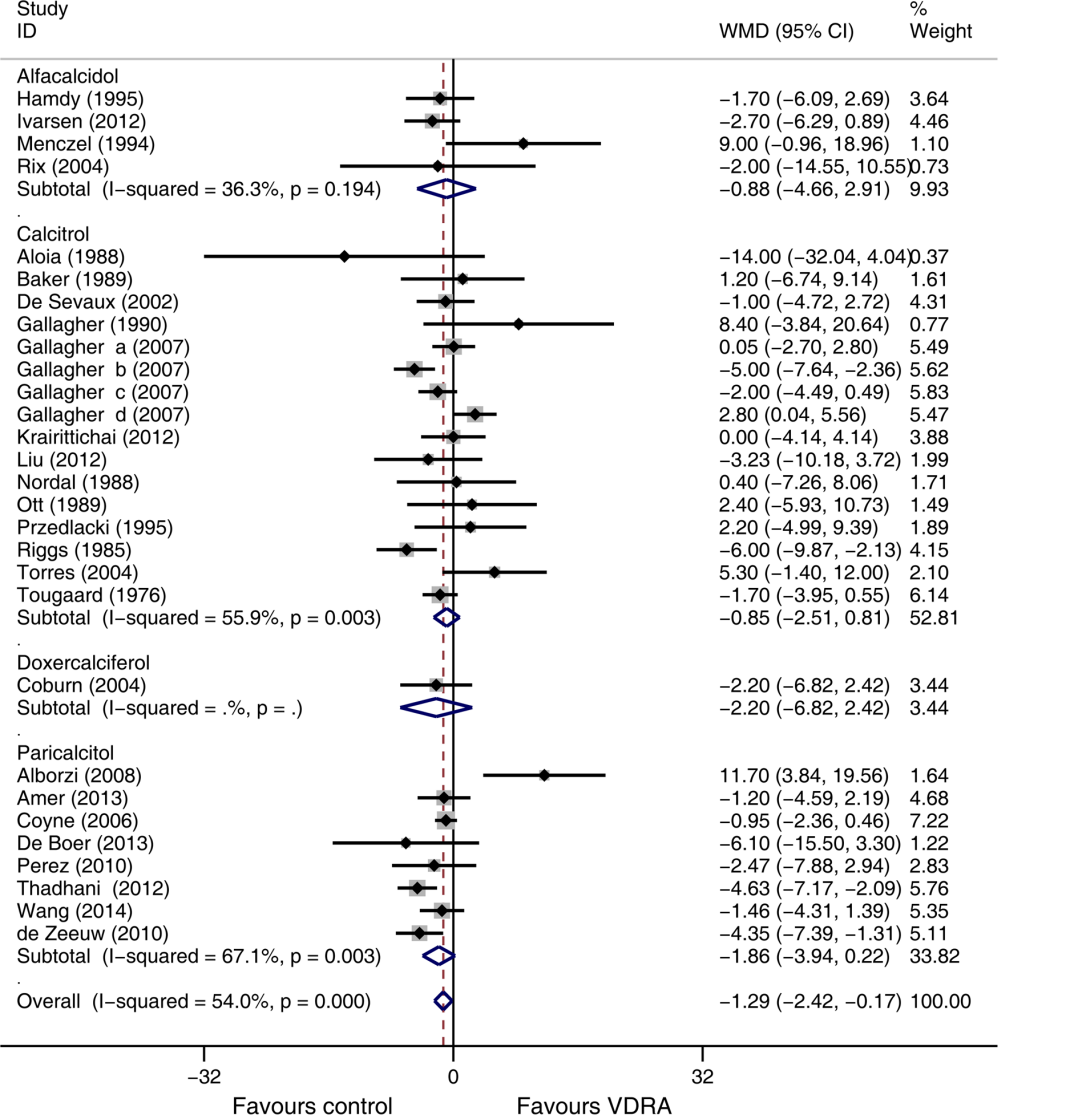
Forest plot comparison of eGFR changes, according to type of vitamin D receptor activators. Weighted mean difference in eGFR (ml/min) in patients who received VDRAs compared with control therapy. Weights are from random effects analysis.

Twenty-six studies [7,10,15,21–29,32–33,35–37,39–43,45–47,48] (comprising 2391 patients) reported eGFR values. Analysis of these studies indicated a slight lower eGFR in the VDRA group than in the control group (WMD -1.29 mL/min/1.73 m^2^, 95% CI -2.42 to -0.17, Fig 2). The heterogeneity across these studies was moderate (*I*^*2*^ = 54.0%, *p* < 0.001). Exclusion of studies with high risk of bias did not change the nature of the association between VDRA use and eGFR. There was no evident publication bias (*p* = 0.24).

Analysis of differences in eGFR according to the individual VDRAs indicated no significant decreases in eGFR in patients randomly assigned to receive alfacalcidol [26–29] (WMD -0.88 mL/min/1.73 m^2^, 95% CI -4.66 to 2.91), calcitriol [32–33,35–37,39–43,45–47](WMD -0.85 mL/min/1.73 m^2^, 95% CI -2.51 to 0.81), doxercalciferol [48](WMD -2.20 mL/min/1.73 m^2^, 95% CI -6.82 to 2.42), or paricalcitol [7,10,15,21–25](WMD -1.86 mL/min/1.73 m^2^, 95% CI -3.94 to 0.22) rather than control treatment (Fig 2).

**Fig 3:**
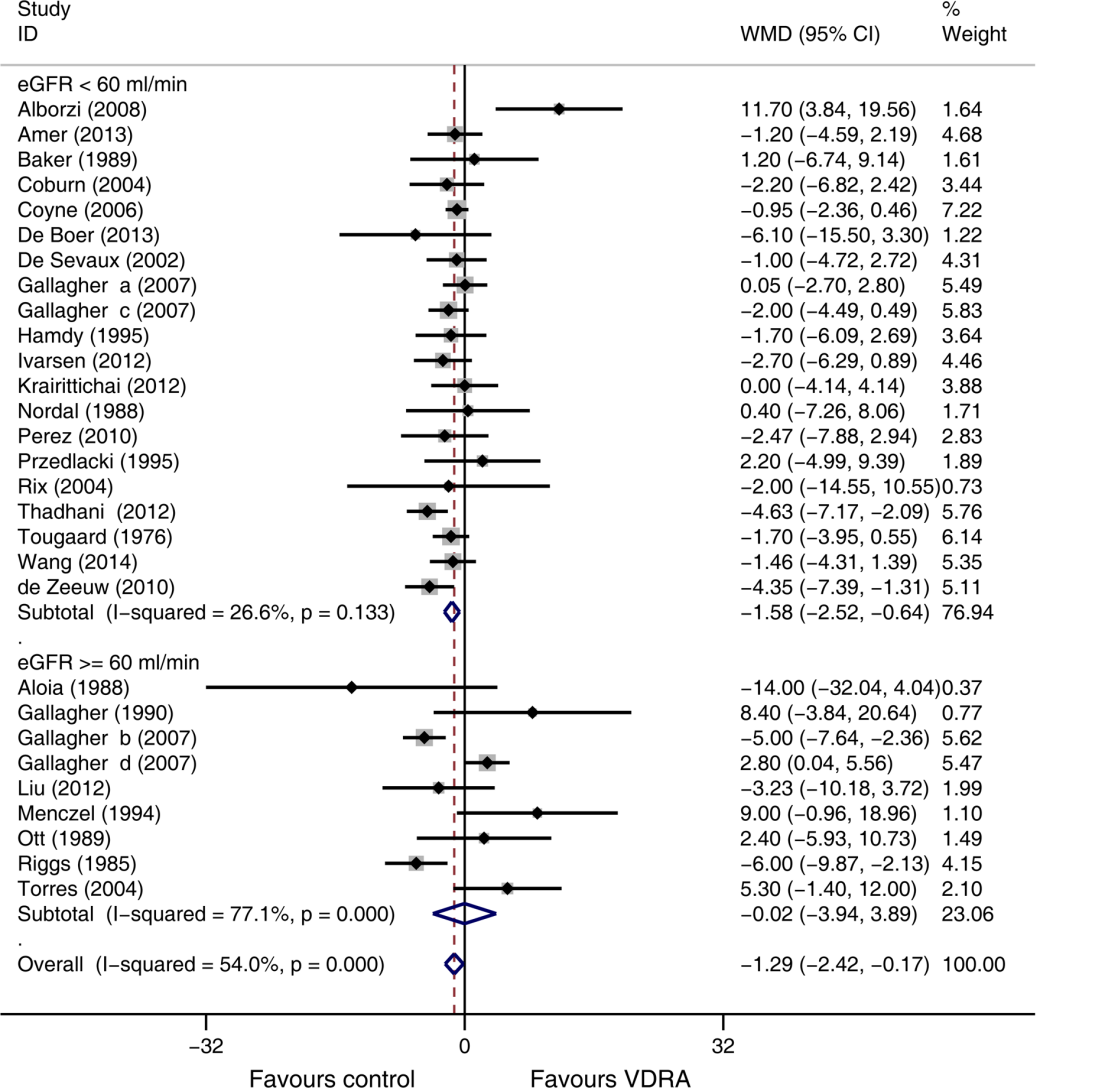
Forest plot comparison of eGFR changes, according to baseline eGFR level. Weighted mean difference in eGFR (ml/min) in patients who received VDRAs compared with control therapy. Weights are from random effects analysis.

Subgroup analysis based on baseline eGFR level indicated a significant difference of eGFR for VDRA patients relative to control patients in the 19 studies [7,10,15,21–26, 28–29,32–33,39–40,43,45,47,48] that enrolled patients with baseline eGFRs lower than 60 mL/min/1.73 m^2^ (WMD -1.58 mL/min/1.73 m^2^, 95% CI -2.52 to -0.64, Fig 3). Meta-regression showed that gender and hypercalcemia were not significantly associated with eGFR decline in VDRAs group (*p* = 0.833 and *p* = 0.302, respectively, see S1 Fig, S2 Fig).

### SCr outcome

**Fig 4:**
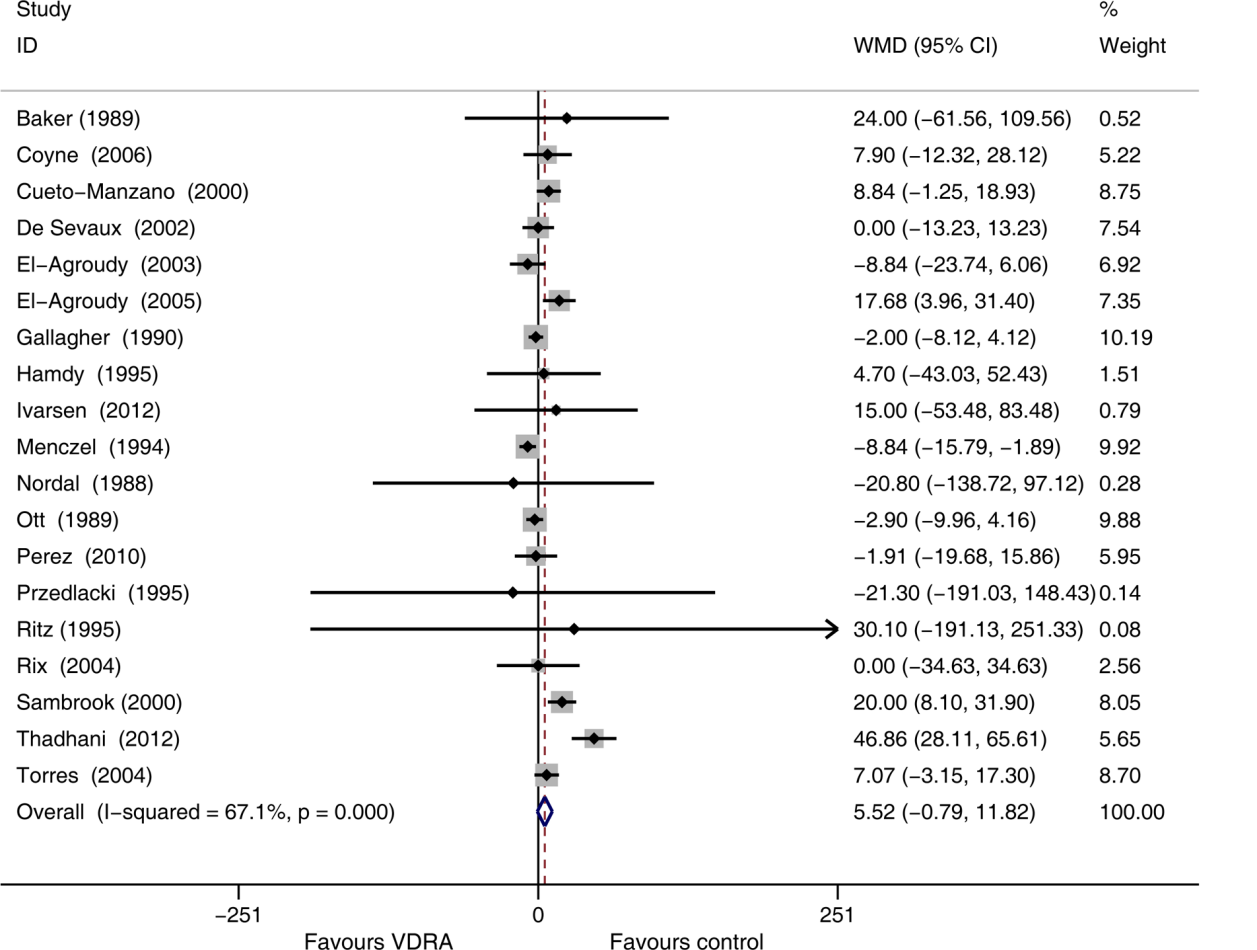
Forest plot comparison of serum creatinine changes for each type of vitamin D receptor activators. Weighted mean difference in serum creatinine (umol/L) in patients who received VDRAs compared with control therapy. Weights are from random effects analysis.

**Fig 5:**
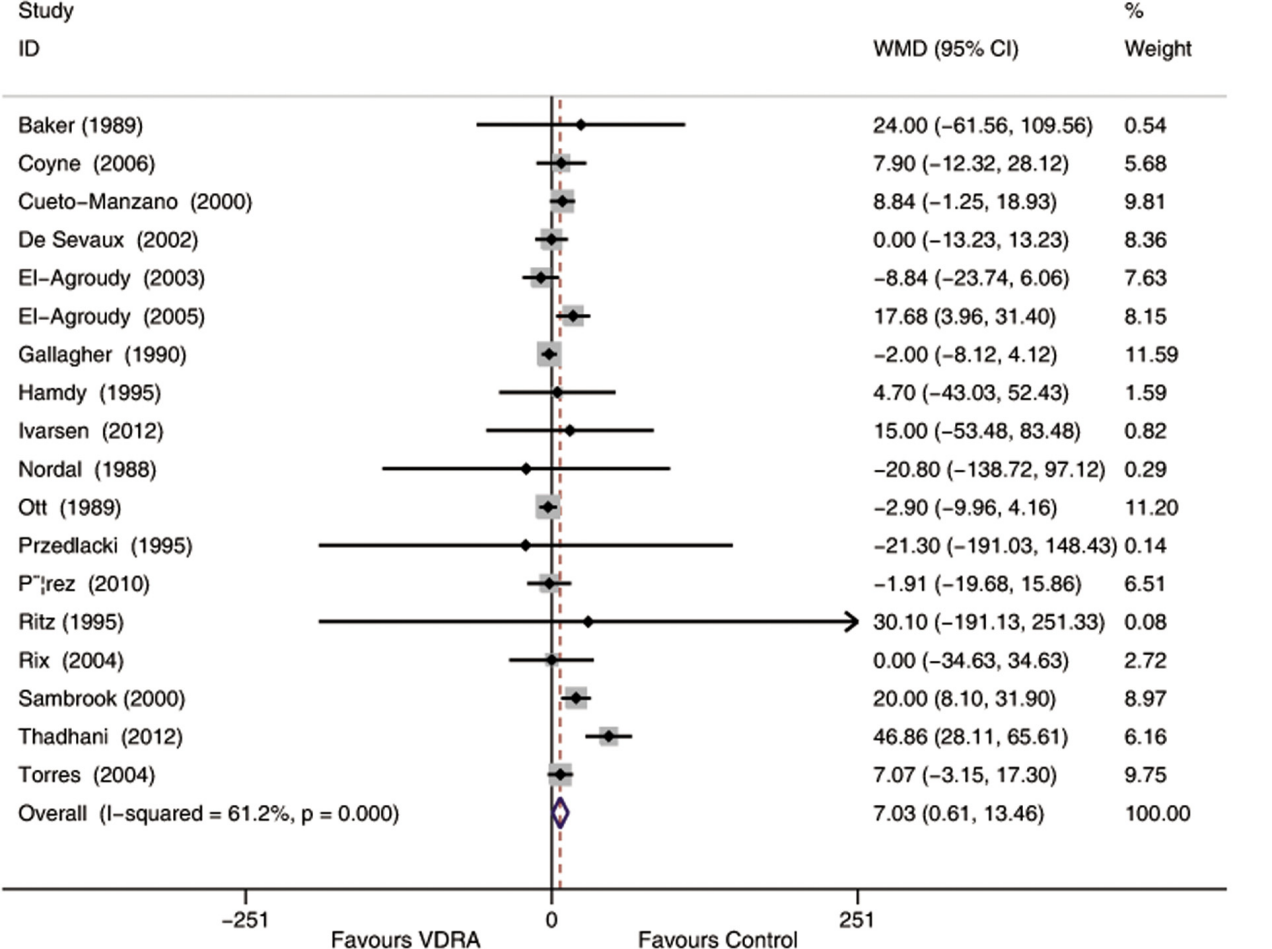
Forest plot comparison of serum creatinine changes by excluding studies with high dropout rates. Weighted mean difference in serum creatinine (umol/L) in patients who received VDRAs compared with control therapy. Weights are from random effects analysis.

Nineteen studies [10,15,23,26–35,38,40–42,44,47](comprising 927 patients) that recorded SCr values reported a slight increase of Scr in VDRA group relative to the control group (WMD 5.52 μmol/L, 95% CI -0.79 to 11.82, Fig 4). Heterogeneity across these studies was moderate (*I*^*2*^ = 67.1%, *p* < 0.001). Publication bias was not evident (*p* = 0.62). Sensitivity analysis by excluding the study [27] with higher dropout rate demonstrated a higher SCr in the VDRAs group than in the control group (WMD 7.03 μmol/L, 95% CI 0.61 to 13.46, Fig 5).

**Fig 6:**
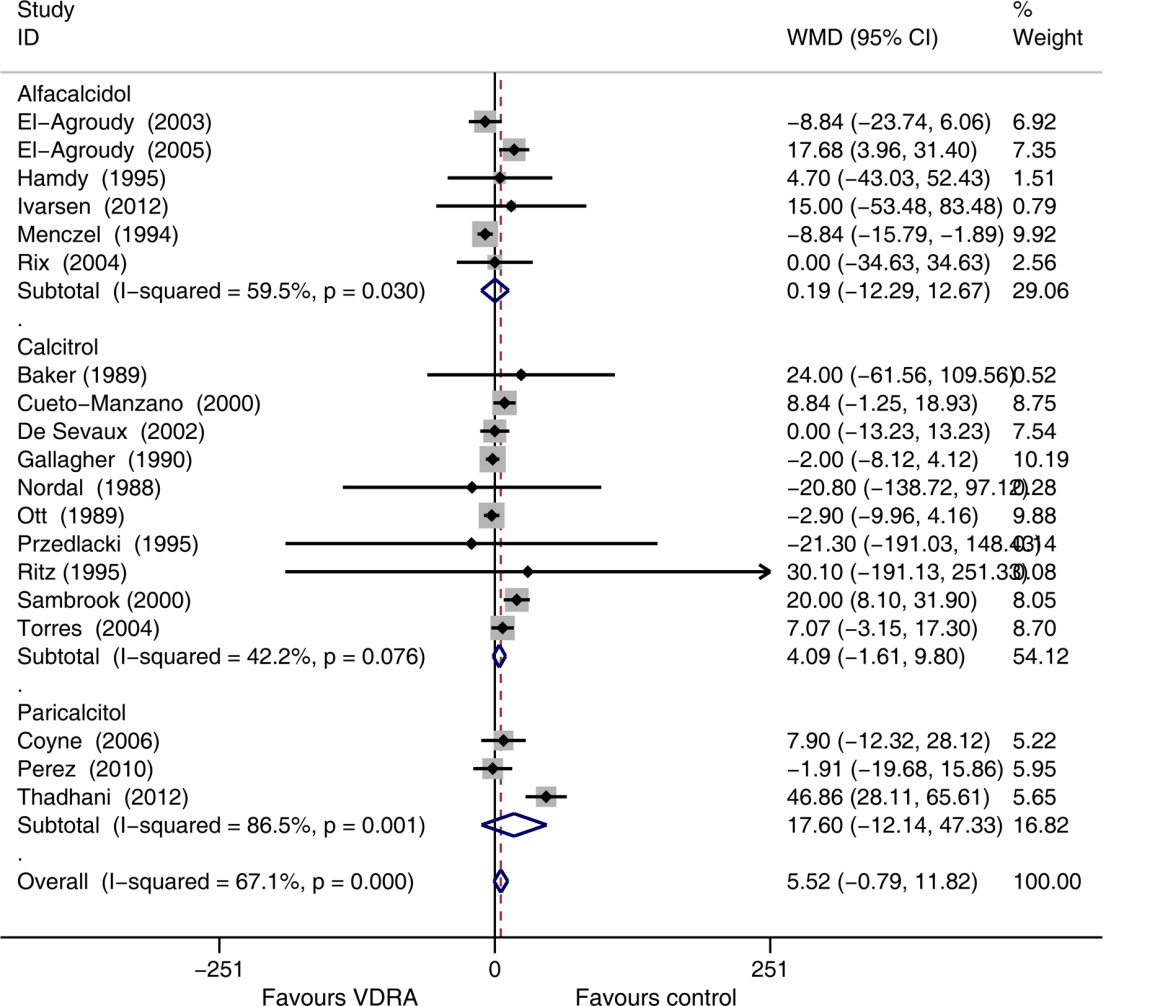
Forest plot comparison of serum creatinine changes, according to type of vitamin D receptor activators. Weighted mean difference in serum creatinine (umol/L) in patients who received VDRAs compared with control therapy. Weights are from random effects analysis.

Subgroup analysis based on the type of VDRAs indicated no significant increase of SCr in patients randomly assigned to alfacalcidol (WMD 0.19 μmol/L, 95% CI -12.29 to 12.67), calcitriol (WMD 4.09 μmol/L, 95% CI -1.61 to 9.80), and paricalcitol (WMD 17.60 μmol/L, 95% CI -12.14 to 47.33) relative to those receiving control treatment (Fig 6).

**Fig 7:**
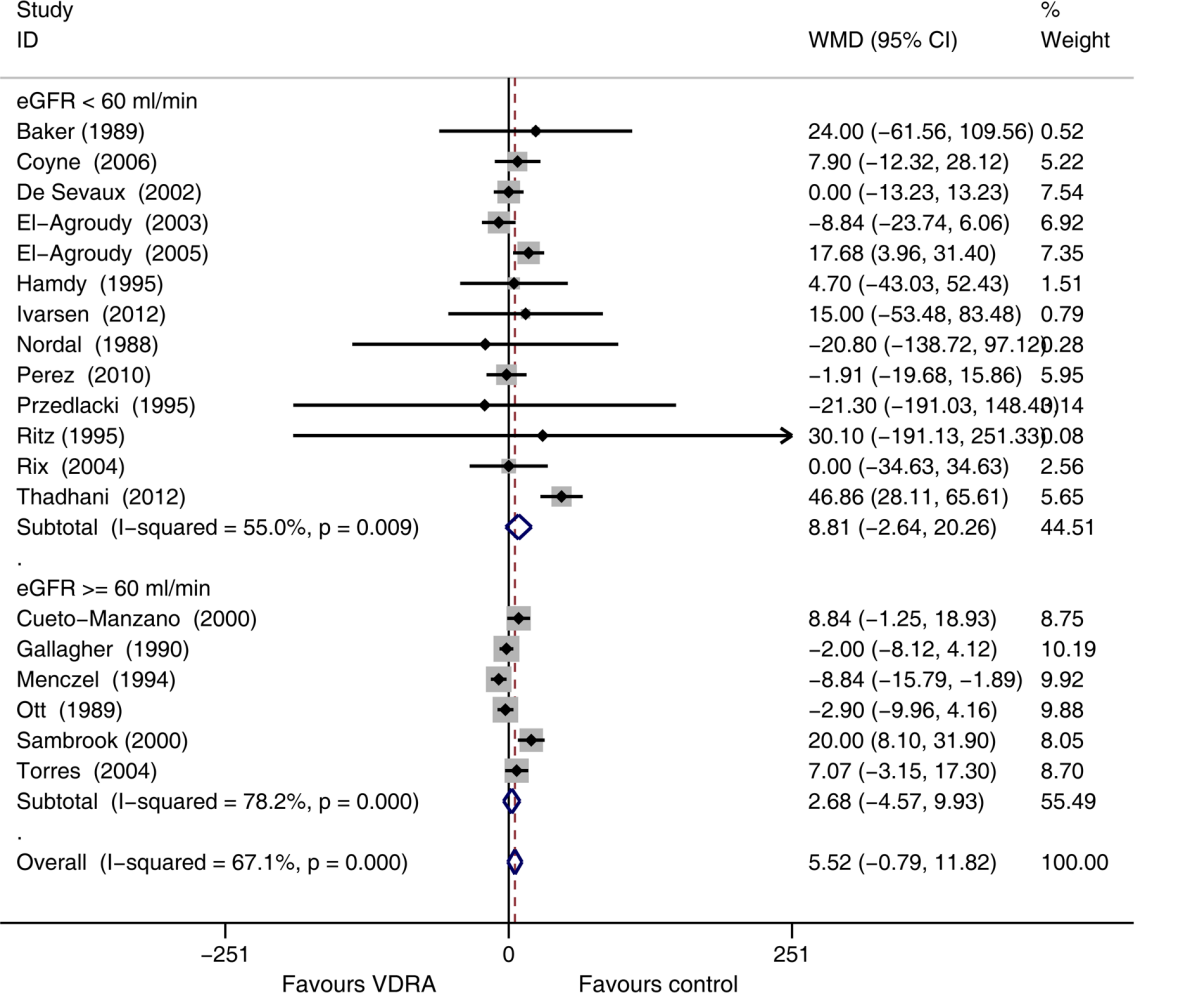
Forest plot comparison of serum creatinine changes, according to baseline eGFR level. Weighted mean difference in serum creatinine (umol/L) in patients who received VDRAs compared with control therapy. Weights are from random effects analysis.

Subgroup analysis based on baseline eGFR level indicated no significant increases of SCr in patients receiving VDRAs among studies that enrolled patients with baseline eGFR values less than or more than 60 mL/min/1.73 m^2^ (Fig 7).

### Other outcomes

Table 2 shows the pooled results for secondary outcomes. Sixteen studies [7,10,15,22–23,25–26,28,30–33,41–43,48] (1753 patients, 18 events) provided data on all-cause mortality. Altogether, mortality was not significantly different in the VDRA and control groups (RR 1.49, 95% CI 0.58 to 3.80; RD 0.00, 95% CI -0.00 to 0.01).

**Table 2:** Results of secondary outcomes. Values are numbers of participants.

Variables	No of studies (references)	VDRAs group	Control group	Relative risk (95% CI)	Risk difference (95% CI)	I^2^ (%)
All-cause mortality	16	12/942	6/821	1.49 (0.58 to 3.80)	0.00 (-0.00 to 0.01)	0
Cardiovascular events	12	15/575	19/452	0.84 (0.42 to 1.71)	-0.00 (-0.03 to 0.03)	3
ESRD	4	14/360	3/263	3.02 (0.91 to 10.09)	0.03 (0.00 to 0.05)	0
Adverse events	18	361/1009	286/849	1.24 (1.04 to 1.47)	0.07 (0.02 to 0.13)	42
Severe adverse events	5	101/491	71/397	1.15 (0.75 to 1.77)	0.02 (-0.07 to 0.12)	56
Hypercalcemia	24	159/1203	40/1037	3.29 (2.02 to 5.38)	0.09 (0.04 to 0.13)	35

VDRAs = vitamin D receptor activators.

CVDs were reported in 12 studies [7,10,21–22,24–25,32,42,44,46–48](1027 patients, 34 events). Again, there was no significant difference in the VDRA and control groups (RR 0.84, 95% CI 0.42 to 1.71; RD -0.00, 95% CI -0.03 to 0.03). However, there was a slight but not significant increase in ESRD among patients receiving paricalcitol rather than control. [7,10,22,48](RR 3.02, 95% CI 0.91 to 10.09; RD 0.03, 95% CI 0.00 to 0.05).

Adverse events occurred in 647 of 1858 patients from 18 studies [7,10,15,21–22,25,32–39,41–44]. Adverse events were slightly more common in the VDRA group than the control group (RR 1.24, 95% CI 1.04 to 1.47; RD 0.07, 95% CI 0.02 to 0.19). However, the pooled RR of severe adverse events after VDRA therapy was comparable that of controls in five studies [7,10,15,22,25] (RR 1.15, 95% CI 0.75 to 1.77; RD 0.02, 95% CI -0.07 to 0.12). Hypercalcemia was reported in 24 studies[7,10,15,21–22,25,27–29,31–39,41–43,45,47–48] (2240 patients, 199 events). Overall, VDRA therapy was associated with a higher risk of hypercalcemia than control therapy (RR 3.29, 95% CI 2.02 to 5.38; RD 0.09, 95% CI 0.04 to 0.13).

## Discussion

This study reviewed existing RCTs to evaluate the effects of VDRAs on kidney function. Ultimately, 31 trials that enrolled a total of 2621 patients met our inclusion criteria. The results indicated a slightly lower eGFR and increase of SCr in the VDRAs group, especially in the sensitivity analysis by excluding studies that had a dropout rate more than 30%. However, subgroup analysis of the 5 studies that not use SCr-based measures did not indicated lower GFR in the VDRAs group.

Precise measurement of GFR is obtained by calculating the urinary or plasma clearance of an exogenous filtration marker, such as inulin, iothalamate, ethylenediaminetetraacetic acid (EDTA), or diethylene triamine pentaacetic acid (DTPA) [49,50]. Among the 31 included studies, one study used the isotope method with ^99m^Tc DTPA [33] and one study used EDTA to measure GFR before and after clinical intervention [45]. Two studies [24,25] calculated GFR by subcutaneous infusion of nonradioactive iothalamate and one study estimated GFR based on measurement of cystatin C [10]. In most of the included studies, the 24-h urinary creatinine and SCr were evaluated for determination of creatinine clearance and eGFR using the Modification of Diet in Renal Disease (MDRD) or Cockcroft-Gault equations.

The main pitfall of using 24-h urinary creatinine clearance for estimation of GFR is the difficulty and potential inaccuracy of urine collection. In particular, this method overestimates GFR by ~10% in individuals with normal renal function, but the overestimation increases to 30% for a patient with low GFR [49]. As an index of GFR, SCr also has limited sensitivity. Some research has examined the effect of VDRAs on serum creatinine generation and clearance. For example, Bertoli et al.[51] showed that treatment with calcitriol for 4 months increased measured SCr and decreased creatinine clearance, but there were no significant changes in measured inulin clearance. Furthermore, SCr fell to the baseline value within 60 days after discontinuation of calcitriol therapy. The authors attributed the increase of SCr to the increased release of creatinine from muscular tissue, probably due to the improvement of uremic myopathy induced by calcitriol. Perez et al.[52] examined the effect of oral calcitriol in treatment of plaque-type psoriasis (baseline creatinine clearance: 103.8 ± 40.1 mL/min/1.73 m^2^). After 6 months, there was a 22.5% decline in creatinine clearance but no significant changes in clearance of inulin or para-aminohippurate (PAH), suggesting that calcitriol altered creatinine metabolism or secretion but did not affect renal function. Recently, Agarwal et al.[9]tested the effect of paricalcitol on SCr in 16 patients with chronic kidney disease (measured GFR: 47.8 ± 17.1 mL/min/1.73 m^2^). The key findings were that short-term paricalcitol treatment led to significant increases in SCr and 24-h urinary creatinine output, but no changes in clearance of creatinine, urea, or iothalamate. Such findings are consistent with the interpretation that VDRA alters creatinine metabolism but does not harm kidney function. In our study, subgroup analysis of the 5 studies that not use SCr-based measures did not indicated lower GFR in the VDRAs group(WMD -0.97 mL/min/1.73 m2, 95% CI -4.85 to 2.92). Hence, it is important to select the most appropriate method to measure renal function in patients taking VDRAs, such as iothalamate or cystatin C.

Vitamin D and its analogs suppress renin expression [53,54], so an increased SCr concentration may have indicated a true decline in GFR, which was seen with use of ACEIs. Thus, we cannot exclude the possibility that VDRAs may have induced or accelerated the progression of renal dysfunction.

Our findings indicated that the VDRA and control groups had no significant differences in the hard endpoints (e.g. all-cause mortality and CVD) and severe adverse events. Episodes of hypercalcemia were more common in the VDRA group than in the control group. In general, treatment with active vitamin D analogs was well tolerated and only a few patients had to stop treatment.

Our study has several strengths, including the use of a comprehensive search strategy (S2 Appendix) and the large study sample. We included all studies that examined the effect of VDRAs on GFR and SCr. This study is the first meta-analysis to assess the effect of VDRAs on kidney function and safety end points. Our study has several limitations. Firstly, most of the included studies were not designed to directly examine SCr or GFR as primary endpoints. Secondly, the dosages of VDRA of the included studies were also different. However, we excluded the study with the highest dosage of calcitriol [45] and the result did not change. Finally, the generalizability of all meta-analyses is limited by protocol heterogeneity and differences among study populations. We attempted to account for heterogeneity by conducting subgroup analysis according to baseline GFR level. This analysis indicated that a VDRA-induced decrease in eGFR was more likely in patients with baseline eGFRs below 60 mL/min/1.73 m^2^. In other words, patients with poor kidney function are more likely to be adversely affected by VDRAs. The treatment durations of the included studies ranged from 1 month to 3 years, a time during which true changes in renal function could occur. Hence, our results should be interpreted with some caution.

In conclusion, the main finding of this systematic review and meta-analysis of RCTs is that VDRAs can lead to elevation of serum creatinine. Future long-duration RCTs with large sample sizes are needed to assess the effects and safety of VDRAs on renal function as the primary endpoint, using non SCr-based measurements.

## Supporting Information

S1 Appendix Means and standard deviations or frequencies of the included studies. (ZIP)

S2 Appendix Search strategy. (DOC)

S1 Fig Meta­regression of eGFR reduction against female proportion (size of circle is proportional to size of trial). (TIF)

S2 Fig Meta­regression of eGFR reduction against hypercalcemia rate (size of circle is proportional to size of trial). (TIF)

S1 Table PRISMA (Preferred Reporting Items for Systematic Reviews and Meta-Analyses) flow diagram of the systematic literature search. (DOC)

S2 Table Risk of bias in included studies. (TIF)

S3 Table Risk of bias in included studies. (TIF)
