# Supplementary material for: Effect of Vitamin D Receptor Activators on Glomerular Filtration Rate: A Meta-Analysis and Systematic Review
Source: PLoS One. 2016 Jan 26;11(1):e0147347. doi: 10.1371/journal.pone.0147347 (PMC4727919; doi:10.1371/journal.pone.0147347)
Supplement: S2 Appendix — (DOC) [file pone.0147347.s002.doc]

Search strategy

EMBASE (OvidSP)

1. Kidney Disease/

2. Kidney Failure/

3. Chronic Kidney Failure/

4. exp haemodialysis/

5. (haemodialysis or haemodialysis).tw.

6. dialysis.tw.

7. (CAPD or CCPD or APD).tw.

8. predialysis.tw.

9. (chronic renal or chronic kidney).tw.

10. (CKD or CKF or CRD or CRF or ESKD or ESRD or ESKF or ESRF).tw.

11. Chronic Kidney Disease/

12. or/1-11

13. (Alfacalcidol or calcitriol or doxercalciferol or paricalcitol).tw.

14. VDRA.tw.

15. (Vitamin D receptor activator).tw.

16. or/13-15

17. (random* OR factorial* OR crossover* OR placebo*).af.

18. exp crossover-procedure/ or exp double-blind procedure/ or

exp randomised controlled trial/ or single-blind procedure/

19. 17 or 18

MEDLINE (Pubmed)

((randomised controlled trial [pt] OR controlled clinical trial [pt] OR randomised [tiab] OR placebo [tiab] OR drug therapy [sh] OR randomly [tiab] OR trial [tiab] OR groups [tiab]) NOT (animals [mh] NOT humans [mh])) AND ((“Kidney Diseases” OR “Chronic Kidney Failure” OR “Kidney Failure” OR “chronic kidney” OR “chronic renal”) OR (CKD or CKF or CRD or CRF or ESKD or ESRD or ESKF or ESRF)) AND ((Alfacalcidol or calcitriol or doxercalciferol or paricalcitol) OR VDRA OR “Vitamin D receptor activator”)
