# Supplementary figures and images for: Effect of Vitamin D Receptor Activators on Glomerular Filtration Rate: A Meta-Analysis and Systematic Review
Source: PLoS One. 2016 Jan 26;11(1):e0147347. doi: 10.1371/journal.pone.0147347 (PMC4727919; doi:10.1371/journal.pone.0147347)

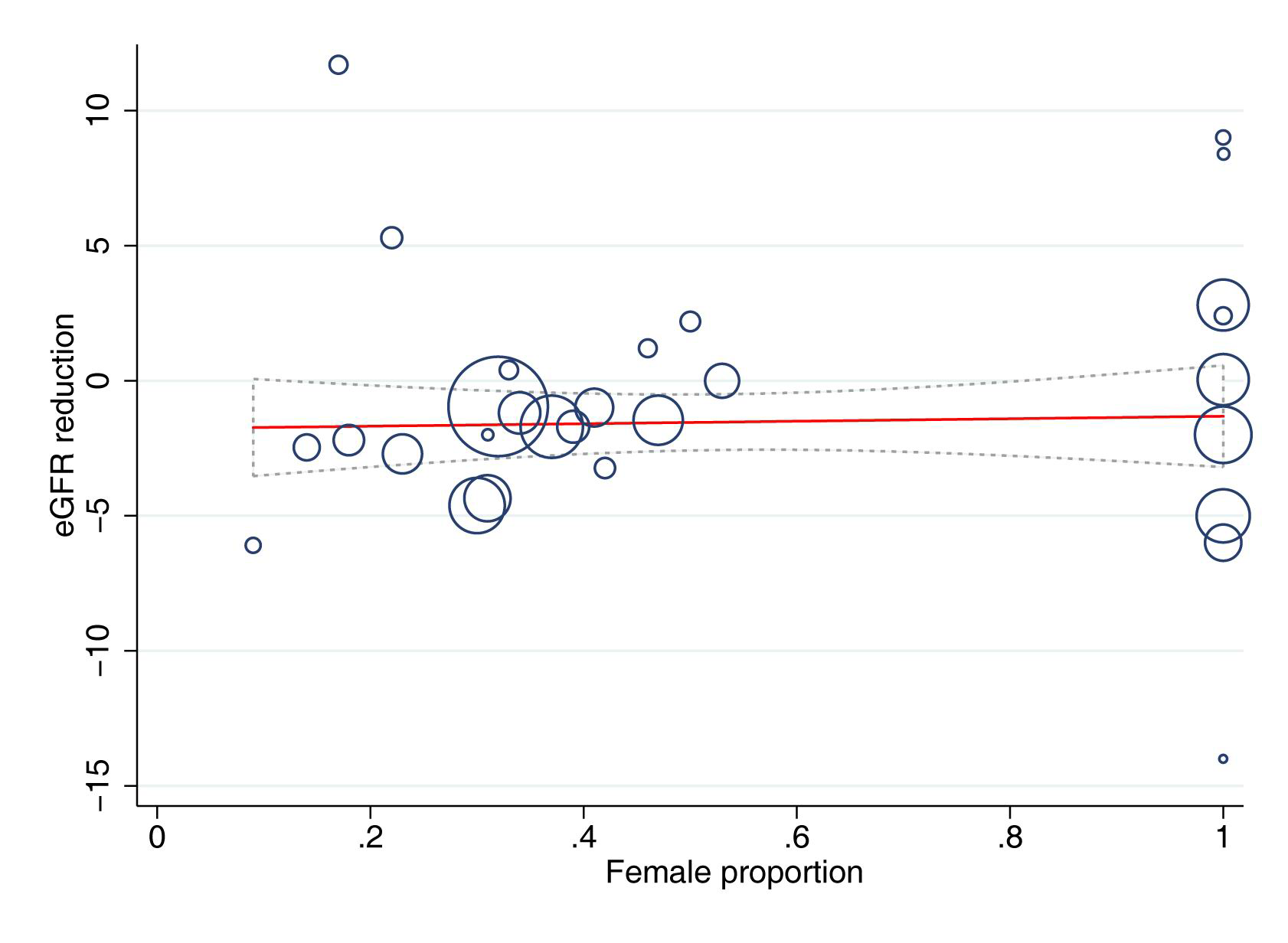

Supplement: S1 Fig — (TIF) [file pone.0147347.s003.tif]

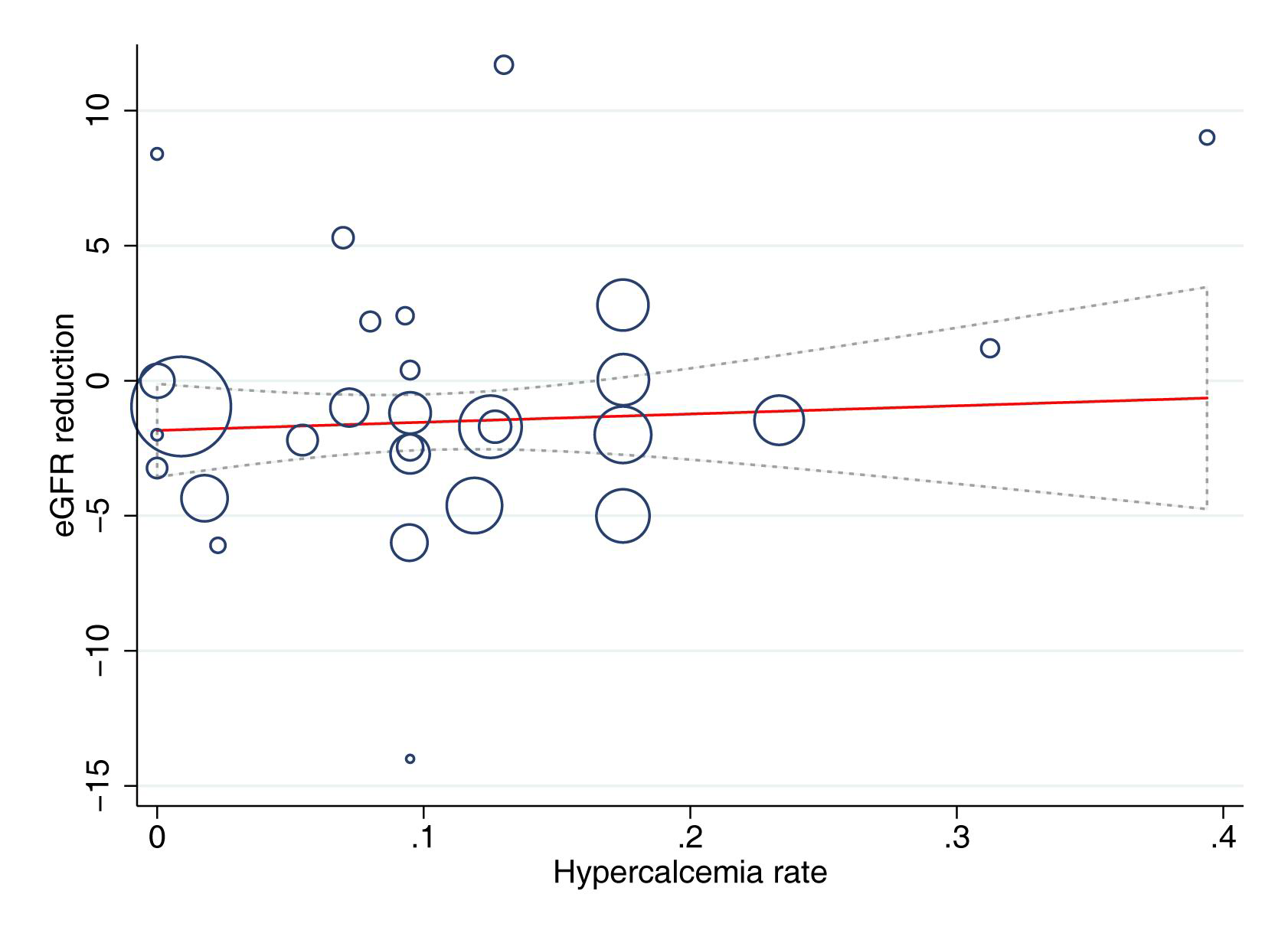

Supplement: S2 Fig — (TIF) [file pone.0147347.s004.tif]

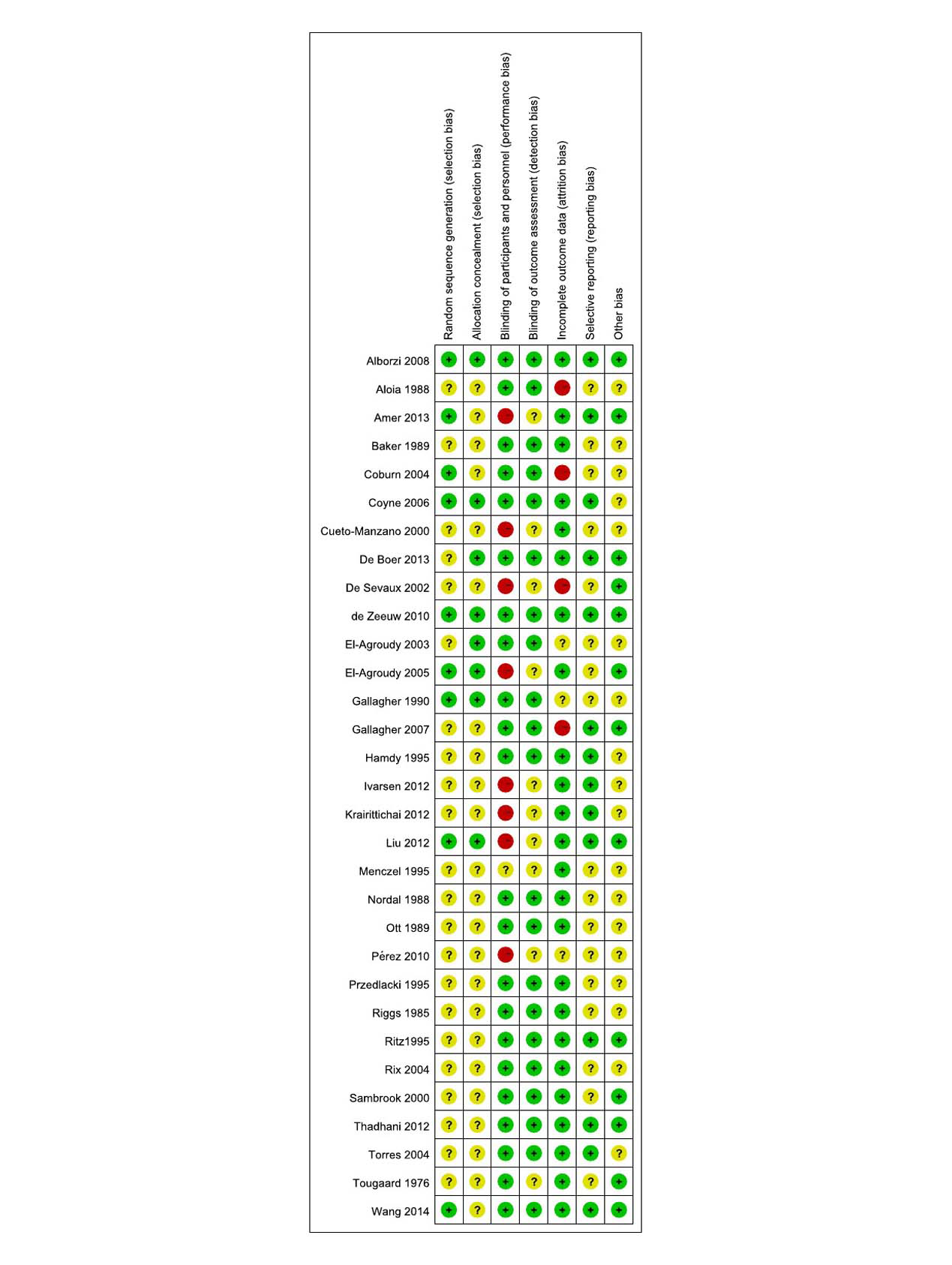

Supplement: S2 Table — (TIF) [file pone.0147347.s006.tif]

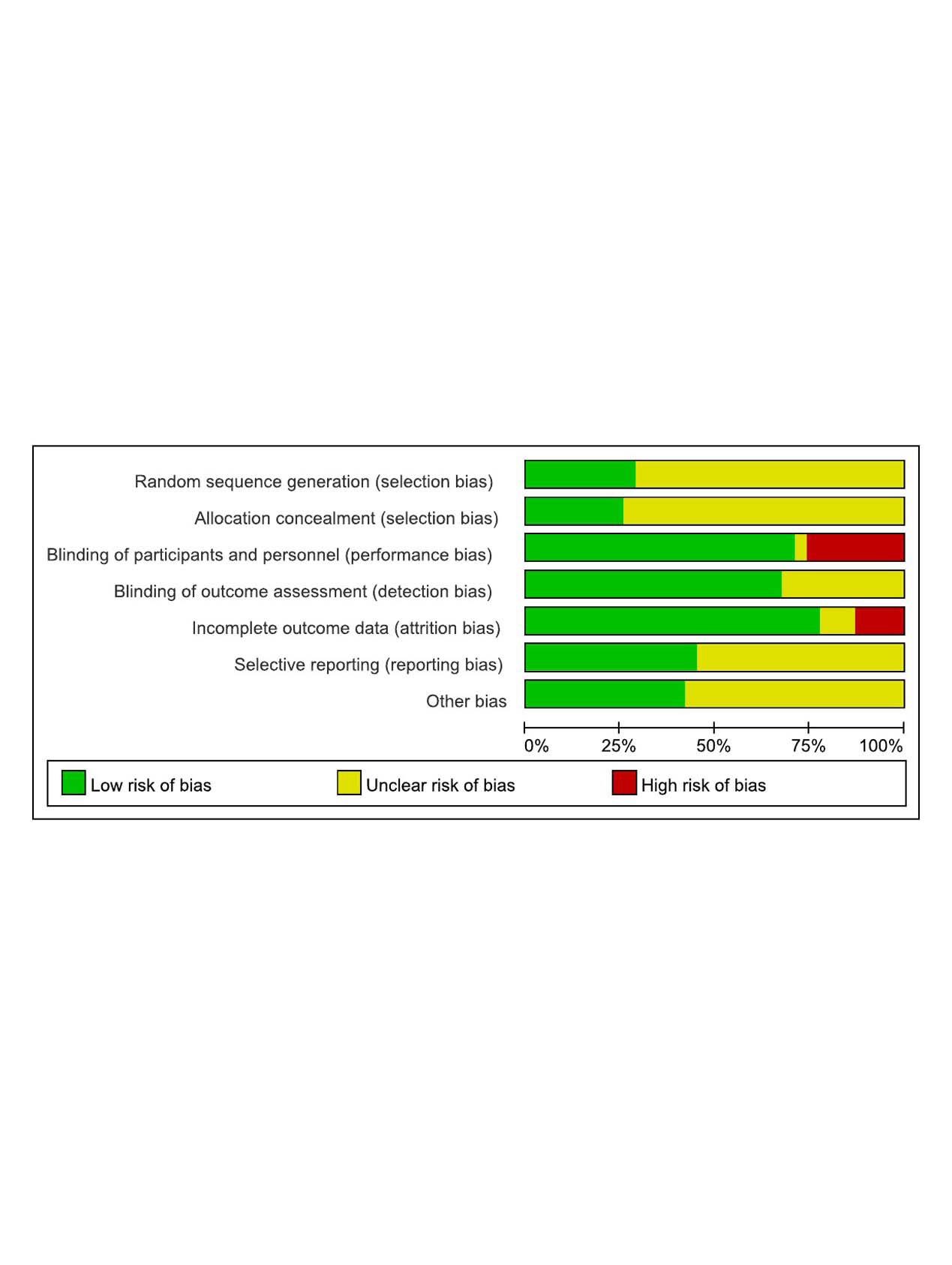

Supplement: S3 Table — (TIF) [file pone.0147347.s007.tif]
